# Simultaneous Determination of Ethanol and Methanol in Wines Using FTIR and PLS Regression

**DOI:** 10.3390/foods13182975

**Published:** 2024-09-19

**Authors:** Vasiliki Thanasi, Ilda Caldeira, Luís Santos, Jorge M. Ricardo-da-Silva, Sofia Catarino

**Affiliations:** 1 LEAF—Linking Landscape, Environment, Agriculture and Food—Research Center, Instituto Superior de Agronomia, Universidade de Lisboa, Tapada da Ajuda, 1349-017 Lisboa, Portugal; jricardosil@isa.ulisboa.pt (J.M.R.-d.-S.); sofiacatarino@isa.ulisboa.pt (S.C.); 2 Associate Laboratory TERRA, Instituto Superior de Agronomia, Universidade de Lisboa, Tapada da Ajuda, 1349-017 Lisboa, Portugal; 3 Instituto Nacional de Investigação Agrária e Veterinária, Polo de Inovação de Dois Portos, Quinta de Almoinha, 2565-191 Dois Portos, Portugal; ilda.caldeira@iniav.pt; 4 MED—Mediterranean Institute for Agriculture, Environment and Development, CHANGE—Global Change and Sustainability Institute, Instituto de Investigação e Formação Avançada, Universidade de Évora, Pólo da Mitra, Ap. 94, 7006-554 Évora, Portugal; 5 Centro de Química Estrutural, Institute of Molecular Sciences, Departamento de Engenharia Química, Instituto Superior Técnico, Universidade de Lisboa, Av. Rovisco Pais 1, 1049-001 Lisboa, Portugal; luis.santos@tecnico.ulisboa.pt; 6 CeFEMA—Research Centre of Physics and Engineering of Advanced Materials, Instituto Superior Técnico, Universidade de Lisboa, Av. Rovisco Pais, 1, 1049-001 Lisboa, Portugal

**Keywords:** wine analysis, ethanol, methanol, FTIR, chemometrics, quality control

## Abstract

Accurate quantification of ethanol and methanol is essential for regulatory compliance and product quality assurance. Fourier Transform Infrared Spectroscopy (FTIR) offers rapid, non-destructive analysis with minimal sample preparation, making it a promising tool for wine analysis. In this exploratory study, the use of FTIR and PLS regression for the simultaneous quantification of ethanol and methanol in wine samples of 11 different Portuguese mono-varietal wines and different vintages deriving from the same winery in Lisbon was investigated. A model was developed, demonstrating the feasibility of FTIR and PLS regression for the simultaneous quantification of ethanol and methanol in wine samples through dedicated models; it showed good prediction capacity for ethanol determination but poorer performance for methanol quantification. The model could be reliable enough for quality control in wine production, but to improve its performance should be enhanced in the future with more samples from different origins, wine types, and a wider concentration range in the case of methanol.

## 1. Introduction

From a broad viewpoint, wine can be described as a mildly acidic hydroethanolic solution, with water and ethanol constituting approximately 97% *w*/*w* of dry table wines. Ethanol is produced by yeast through the fermentation of hexose sugars, namely fructose and glucose [1]. The ethanol levels in wine are regularly measured to monitor alcoholic fermentations, usually through density measurements, to maintain quality standards and comply with legal regulations. Many countries and wine regions have set minimum ethanol concentration requirements for a beverage to be considered wine, and tax regulations might also be tied to ethanol concentration [2]. According to the basic definition of the International Organisation of Wine and Vine (OIV), wine is considered a beverage resulting exclusively from the partial or complete alcoholic fermentation of fresh grapes, whether crushed or not, or of grape must, with an actual alcohol content not less than 8.5% vol. and up to a maximum of 15% vol. for table wines and 22% vol. in fortified wines [3]. In typical table wines, this alcohol content consists of ethanol, with minor contributions from other higher alcohols and a small amount of methanol [2]. The alcoholic strength of wine is determined by its alcohol content, which is expressed as a percentage by volume [4].

Ethanol, as the primary volatile compound in wine with antiseptic properties, significantly impacts the sensory perception of aromatic attributes and the detection of other volatile compounds. As a result, alcohol plays a crucial role not only in the sensory experience of wine but also in its interactions with other components such as aromas and tannins. These interactions also influence the viscosity and body of the wine, as well as the perceptions of astringency, sourness, sweetness, bitterness, aroma, and flavor [5,6]. In recent years, a phenomenon of wines with higher alcoholic content has been witnessed due to several factors related to the increase in sugar levels in musts, caused by climate change [7]. Climate change, driven by rising global temperatures, is impacting the alcoholic content of wines by accelerating grape ripening and increasing sugar levels in the fruit, which in turn leads to higher alcohol levels during fermentation [8]. This can alter traditional styles and challenge the production of certain wine types like ice wines [9]. On the other hand, there is a rising demand from consumers in many countries for beverages with lower alcohol (9–13% vol.) [10,11], driven by health and social concerns [12,13,14].

Methanol can be produced naturally in wine, both before and during alcoholic fermentation, mainly as a result of the breakdown of pectins by pectinase enzymes (such as pectin methylesterase) [15]. Wines fermented with grape skins tend to have higher levels of methanol, which explains why red wines typically have higher levels than rosé or white wines [16]. The presence of methanol in wine is influenced by various factors, including grape variety (especially grape skins, which have a high pectin content), grape sanitary state, maceration conditions, fermentation temperature, and the use of pectolytic enzymes or the pre-bottling antiseptic dimethyl dicarbonate (DMDC) [17].

Methanol is known to be one of the most harmful components in alcoholic drinks. The International Organisation of Vine and Wine (OIV) has set maximum acceptable limits for methanol content in wines: less than 400 mg/L for red wines and less than 250 mg/L for white or rosé wines [18]. However, from a toxicological perspective, the established methanol limits for wine do not pose significant safety concerns, as methanol is present in wine at relatively low levels. The determination of methanol content in wine is significant as it reflects its origin and is associated with the establishment of regulatory limits. These limits are important from a technological standpoint as they relate to wine quality and proper fruit handling during harvest and subsequent processing [17].

Considering all the above, in wine production, accurately measuring these two key parameters is essential to ensure the quality and safety of the final product and meet consumer demands. Traditionally, electronic densimetry is used to determine alcoholic strength, while gas chromatography with flame ionization detection (GC-FID) is the preferred method for methanol determination [18]. However, these techniques are labor- and time-intensive, leading many wineries and laboratories to adopt instrument-based techniques. FTIR, combined with specialized software for grape and wine analysis, is gaining attention for its non-destructive, rapid, and automation-friendly approach [19,20]. This study aims to explore the potential of FTIR combined with chemometrics for quantifying methanol in the presence of ethanol in wine samples.

## 2. Materials and Methods

The feasibility of using FTIR to quantify ethanol and methanol in wine samples simultaneously was tested using standard solutions of ethanol in water, methanol in water, and various combinations of both. A mathematical model was developed using Partial Least Squares (PLS) regression. Two reference methods were employed to independently validate the model: electronic densimetry for ethanol determination and GC-FID for methanol determination.

### 2.1. Materials

#### 2.1.1. Reagents

Highly pure methanol (Merck, Darmstadt, Germany, 99.8% purity) and ethanol (Merck, Darmstadt, Germany, 99.9% purity) were used for the preparation of standard solutions of ethanol in water (0–25% *v*/*v*) and methanol in water (0.1–1.4% *v*/*v*).

#### 2.1.2. Wine Samples

Different white and red wines from distinct grapevine varieties (*Vitis vinifera* L.) and different vintages were used, namely ‘Cabernet Sauvignon’ (2019, 2018, 2016), ‘Trincadeira’ (2019, 2016), ‘Touriga Nacional’ (2019, 2018, 2016), ‘Syrah’ (2019, 2018, 2016), ‘Moscatel de Setúbal’ (2019, 2016), ‘Arinto’ (2019), ‘Viosinho’ (2019), ‘Alvarinho’ (2019, 2016), ‘Moscatel Galego’ (2019), ‘Encruzado’ (2019), and ‘Macabeo’ (2019). The wines were produced using grapes grown in Tapada da Ajuda vineyards, at the winery of Instituto Superior de Agronomia, following traditional white and red winemaking processes.

#### 2.1.3. Sample Preparation

Before analysis, the samples underwent distillation for both densimetry and GC-FID to minimize matrix effects and ensure accurate measurements according to [18]. However, for FTIR, the samples were used directly without prior distillation, employing chemometrical approaches to account for the matrix effects. Densimetry, used for ethanol quantification, can be influenced by matrix components like sugars, acids, and other alcohols, which may alter the density [21], while GC-FID, employed for both ethanol and methanol quantification, can be affected by the co-elution of volatile compounds, leading to potential inaccuracies [22,23]. To minimize volatilization during sample preparation, both standards and samples were placed in airtight vials and handled quickly in a temperature-controlled environment.

### 2.2. Equipment and Conditions

#### 2.2.1. FTIR Measurements

Duplicate FTIR measurements took place in transmission mode with the use of a Perkin Elmer LQATM 300 FT-IR wine analyzer. The instrument consists of a standard, high-performance, room-temperature MIR detector. Infrared spectral data were collected in the range of 950–4000 cm^−1^ with 4 scans and a resolution of 0.5 cm^−1^. A liquid handling system was designed to deliver the sample to the liquid cell to perform the measurement. Calcium fluoride (CaF_2_) windows were used on the liquid cell. Cleaning and zeroing solutions provided by the manufacturer (Perkin Elmer, Inc., Waltham, MA, USA) were also automatically pumped into the cell. The zeroing solution used for the background measurements was a buffering agent replicating the pH and ionic strength of the wine, without containing the analytes under study.

#### 2.2.2. GC Measurements

Methanol content was determined by gas chromatography (GC) on distilled wine samples by adding an internal standard according to the “Portuguese Official Standards for Spirits and Alcoholic Beverages”, NP 3263, 1990 [24]. A distilled sample of 10 mL was mixed with 1 mL of 4-methyl-2-pentanol solution (1 g/L) as the internal standard, and 1 microliter of the mixture was injected in triplicate into a Focus GC gas chromatograph (Thermo Scientific, Waltham, MA, USA). The GC was equipped with a flame ionization detector (FID) set at 250 °C; a fused silica capillary column of polyethylene glycol (DB-WAX, JW Scientific, Folsom, CA, USA) 60 m in length, 0.32 mm i.d., and with 0.25 µm film thickness; and an injector set at 200 °C operating in split mode (split ratio 1:6). The carrier gas was hydrogen (3.40 mL/min). The oven temperature program was 35 °C (for 8 min), then increased at 10 °C/min until 200 °C and held at this temperature for 9 min. The quantification was performed by analyzing hydroalcoholic methanol solutions in similar conditions.

### 2.3. Data Analysis

A Partial Least Squares (PLS1) regression model was developed to correlate spectral data with ethanol and methanol concentrations in wine. The model was calibrated using 35 standard solutions (ethanol: 0–20% *v*/*v*, methanol: 0.04–3.2 g/L) and pre-processed. The full spectral range (950–4000 cm^−1^) was utilized, applying Standard Normal Variate (SNV) Detrending for noise reduction. Deconvolution was performed to reduce interference between overlapping spectral features, followed by the application of the 4th derivative with 13 data points to further enhance spectral resolution. Model stability was evaluated by analyzing both spectral and concentration outliers, and cross-validation was performed to assess prediction accuracy. External validation of the model also took place. The predicted ethanol and methanol concentrations of 20 wine samples were compared to reference measurements using regression analysis to establish correlations between the new method and reference methods. The data analysis was conducted using Perkin Elmer Spectrum IR Version 10.6.2 software.

## 3. Results

### 3.1. Identification of Spectra Modifications

**Figure 1:**
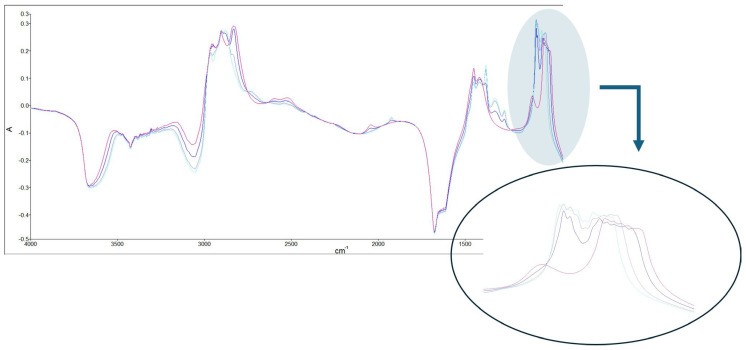
FTIR spectra of pure ethanol, pure methanol, and mixtures of ethanol and methanol.

To identify the spectra modifications for methanol and ethanol, pure ethanol, pure methanol, and mixtures of ethanol and methanol in two different ratios—(*v*:*v*) 9:1 (11.1% methanol in ethanol) and 1:1 (50% methanol in ethanol)—were used (total volume of 10 mL). The region of the IR spectra between 4000 and 2500 cm^−1^ is particularly affected by the contributions of the OH stretching of H_2_O, which overlap with the OH stretching modes originating from the organic molecules [25,26]. Small differences observed between 3700 and 3000 cm^−1^ mostly reflect the different water content of the reference solutions. As illustrated in Figure 1, the most remarkable changes occurred in the spectral region 1000–1200 cm^−1^. 

**Figure 2:**
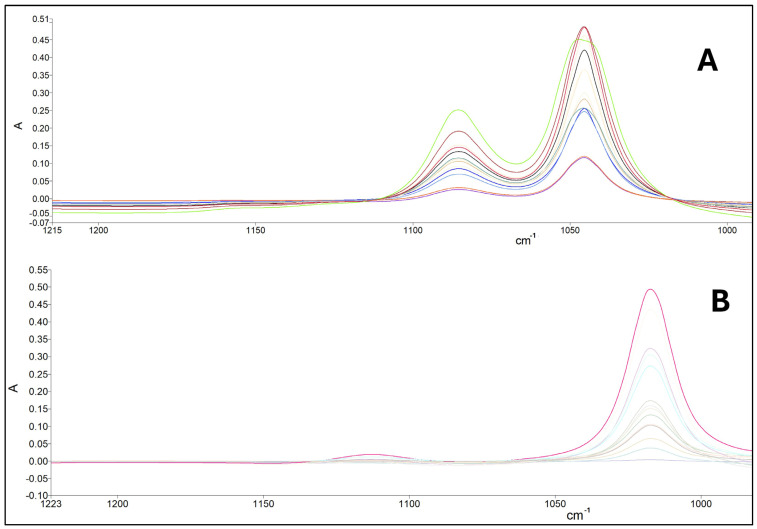
FTIR spectra for different concentrations of (**A**) ethanol standards and (**B**) methanol standards.

Moreover, standard solutions of ethanol in water (0–25% *v*/*v*) and methanol in water (0.1–1.4% *v*/*v*) were used to identify the respective characteristic bands. According to Figure 2, ethanol solutions presented characteristic vibration frequencies at 1047 cm^−1^ (major signal) and 1087 cm^−1^ (minor signal), while methanol had characteristic frequencies at 1020 cm^−1^ (major signal) and 1112 cm^−1^ (minor signal). These frequencies are specific to stretching vibrations of C-O bonds in these molecules [27].

Different standard solutions of methanol and ethanol were used to build a calibration curve based on Beer–Lambert’s law [28], showing a good correlation for ethanol (correlation: 0.989, Standard Error: 1.349, Standard Error of Prediction: 1.656) and methanol (correlation: 0.999, Standard Error: 0.021, Standard Error of Prediction: 0.021).

**Figure 3:**
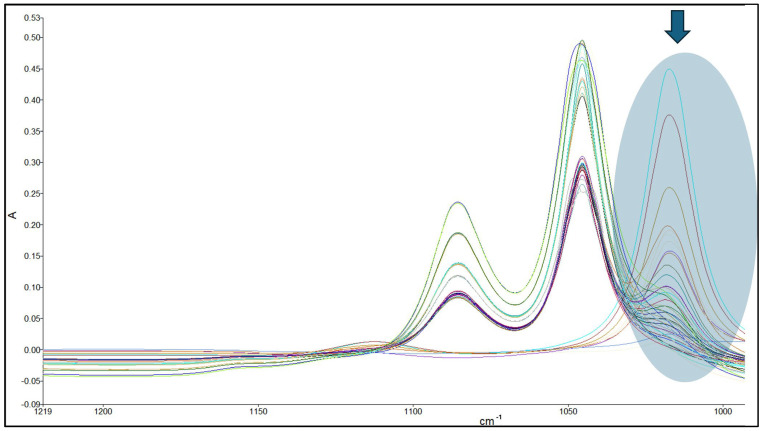
FTIR spectra for different concentrations of ethanol–methanol solutions in water.

However, when considering solutions of both methanol and ethanol in water, Beer–Lambert’s law did not give satisfactory results. When using different ratios of ethanol and methanol mixtures, such as 9:1 and 1:1, we observed changes in absorbance at specific bands. Increasing the amount of methanol in the mixture led to decreases in absorbance at the 1047 and 1087 cm^−1^ bands and increases in absorbance at the 1020–1030 cm^−1^ and 1112 cm^−1^ bands, as expected. However, there is some overlap of the bands (Figure 3). Beer–Lambert’s law requires clear and isolated absorbance for accurate quantification, so it was not possible to use it. As a result, a specific range should be used to accurately measure methanol and ethanol concentration in wine samples rather than relying on a single characteristic band. Multivariate techniques and the development of a PLS model were necessary for this purpose [29]. Standard solutions containing both ethanol and methanol were used to develop the model, with concentration ranges of 0–20% *v*/*v* for ethanol and 0.04–3.2 g/L for methanol (and all their possible combinations) based on their typical levels in wine samples.

**Figure 4:**
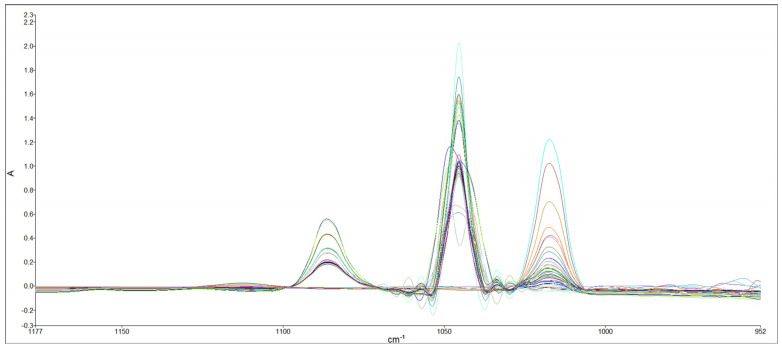
FTIR spectra for different concentrations of ethanol–methanol solutions in water after deconvolution.

**Figure 5:**
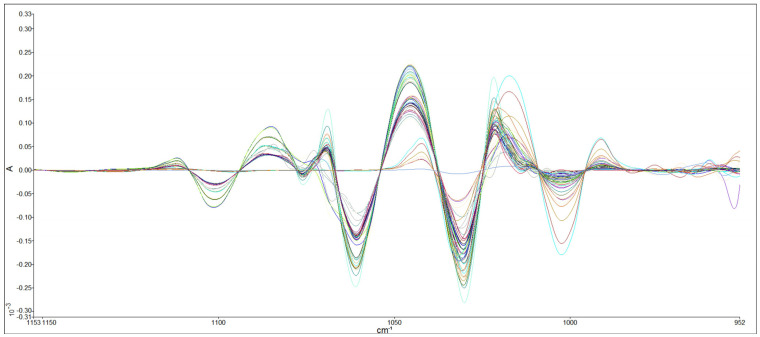
FTIR spectra for different concentrations of ethanol–methanol solutions in water after deconvolution and derivative process.

It can be challenging to simultaneously determine the levels of methanol and ethanol in wine samples due to the low levels of methanol and the proximity of their characteristic bands [30], as shown in Figure 3. To address this issue, a pre-treatment process called deconvolution was employed. Deconvolution involves a line-narrowing process to reduce interference between unresolved features. It is used to estimate the positions and intensities of overlapping absorption bands [31]. The FTIR spectra of the ethanol and methanol mixtures after the deconvolution process are presented in Figure 4. After the deconvolution process, the fourth derivative order with 13 data points of calculation was also applied (Figure 5).

Derivative curves usually have sharper features than the original spectra, which enables them to reduce the effects of overlapping bands and suppress background effects. The derivative process uses the Savitzky–Golay procedure to estimate the derivative of a smooth curve, constructed through the original data points of the original spectrum. Also, it uses a number of neighboring data points to estimate the curve. As the number of data points used in the calculation increases, the contribution of broader features increases relative to narrow features [32].

Simultaneously quantifying ethanol and methanol in wines using reference methods also presents challenges. Ethanol is usually present in much lower concentrations, and its effect on the overall density of a wine is minimal and indistinguishable from other components in the matrix [1]. Therefore, densimetry cannot differentiate between ethanol and methanol or provide accurate measurements of methanol in the presence of ethanol. GC-FID also presents difficulties due to ethanol’s significantly higher concentration compared to methanol. The high ethanol concentration can lead to column saturation, where the stationary phase becomes overloaded, resulting in poor separation of methanol from ethanol. Additionally, the flame ionization detector (FID) might experience saturation, as the strong signal from ethanol can overwhelm the detector, reducing its ability to accurately measure the lower concentrations of methanol. These factors complicate the simultaneous and accurate quantification of both compounds [18,24].

### 3.2. Development of the PLS Regression Model

These spectra were used to develop a PLS regression model. PLS1 is an algorithm in which each property is analyzed individually with respect to the spectral data. PLS seeks to express the variation in the property information by correlating it with the spectral information. The spectra are modeled by a different set of factors for each property, and the concentration values are modeled by the respective factors. As a result, it contains separate calibrations equal to the number of properties in the method.

#### 3.2.1. Standards

For the development of the model, 35 standard solutions of ethanol and methanol in water in different concentrations were used (0–20% *v*/*v* ethanol range and 0.04–3.2 g/L methanol range) after the previous pre-treatment procedures. The pre-treatment was conducted using Perkin Elmer Spectrum IR Version 10.6.2.

#### 3.2.2. Pre-Processing

For the development of the model, the full range was used (950–4000 cm^−1^), along with an instrument response weighting and the use of Standard Normal Variate Scaling (SNV) Detrending.

#### 3.2.3. Model Review

After the calibration of the model with the use of Perkin Elmer Spectrum Quant Version 10.6.2, final regression data were obtained, which are summarized in Table 1.

The model is considered stable because, in both graphs of the estimated values in relation to the specified values for ethanol and methanol, the standards appear on the regression line for the concentrations tested.

The outlier graphs display two cutoff lines on both axes. The vertical cutoff represents spectral outliers and the horizontal cutoff represents concentration outliers. Both spectral outlier tests for ethanol and methanol were successful, as no standard appeared to exceed the cutoff. Regarding the concentration outliers, both of them presented two concentration outliers. Finally, considering the values of Standard Error of Prediction (SEP) for both properties and analyzing the relative graphs representing the effect of principal components’ PCs on the SEP, we conclude that the model can predict the values of independent samples with a relatively low error rate.

### 3.3. Independent Validation

**Figure 6:**
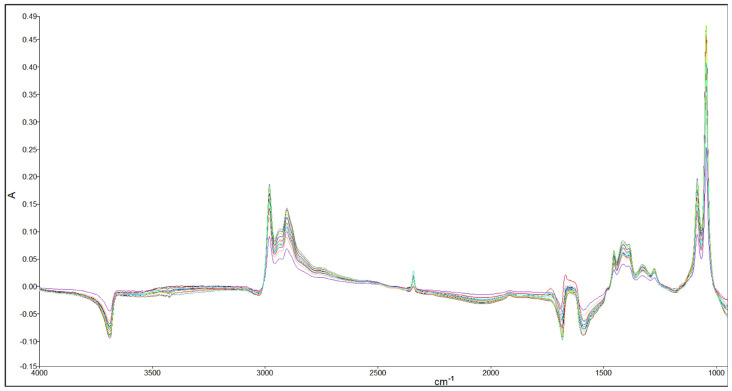
FTIR spectra for the 20 different wine samples.

For an external validation of the model, the ethanol and methanol levels of 20 wine samples were identified [Figure 6]. The reference method used for ethanol determination was electronic densimetry, and for methanol, gas chromatography with flame ionization detection (GC-FID) was used. The measurements took place in duplicates, and the results are shown in Table 2.

The ethanol and methanol content of the 20 wine samples previously described was predicted by the developed model. To compare a new analytical method with a reference method, the typical approach involves constructing a regression line that plots the results of the new method against those obtained by the reference method [33]. The results showed a good correlation with the reference values for the ethanol determination (y = 0.9557x, R^2^ = 0.988). However, the model did not show satisfactory prediction capacity for the methanol content. This was attributed to the poor sensitivity of FTIR in quantifying low concentration levels, as most of the samples exhibited methanol concentrations very close to zero. FTIR is better at determining compounds with a concentration higher than 1 g/L due to its ability to engage absorption phenomena [19,20]. Moreover, keeping in mind that for the reference methods, the determination was carried out with the wine distillate, direct FTIR measurement can be considered more affected by the matrix effect [34] (see Section 2.1.3).

## 4. Conclusions

Methods developed based on FTIR measurements in winemaking are usually focused on specific parameters or chemical compounds or are applied to a particular stage of the winemaking process. However, simultaneously determining multiple parameters has proven to be quite challenging in most cases. As FTIR combined with chemometrics is already used for routine analysis in wine production, there is a growing interest in developing a new chemometric model that can identify both ethanol and methanol.

The simultaneous determination of methanol and ethanol content in wine production is crucial for several reasons. It allows for efficient quality control, ensuring that both compounds are within safe and acceptable limits. This is critical for maintaining wine quality and consumer safety. This dual measurement is also vital from a technological standpoint, as it reflects proper fruit handling during harvest and processing, directly impacting the final product’s quality. By simultaneously monitoring both methanol and ethanol, winemakers can optimize production processes, ensure regulatory compliance, and detect potential adulteration, all while improving overall efficiency and cost-effectiveness.

In our study, the wine samples were obtained from the same winery but from different grapevine cultivars and harvests, without including other types of wines. This limited sample diversity may result in poor representation. Additionally, the complexity of the wine matrix and the chemical similarity of the compounds under study make interpreting the spectra quite challenging. Also, comparing a single method for the simultaneous determination of two parameters with two different reference methods for the individual determination of the two parameters can lead to inaccurate results. Therefore, simultaneously determining both properties in this study presents higher difficulty.

In conclusion, the developed model demonstrates the feasibility of using FTIR for the simultaneous quantification of ethanol and methanol in wine samples through dedicated models. The model could be reliable enough for quality control in wine production. However, in the future, the model should be enhanced with more samples from different wine types and origins, including other grapevine cultivars and different winemaking technologies from various wineries. For better prediction capacity in methanol determination, another set of samples with different and higher levels of methanol is required.

## Figures and Tables

**Table 1:** Regression model summary.

Property	Number of PCs	%Variance (R Squared)	Std. Error of Estimate (SEE)	Std. Error of Prediction (SEP)	Cross-Validation SEP	Mean Property Value
Ethanol	10	99.9977	0.03447	0.03879	1.33	12.5
Methanol	9	99.9183	0.03799	0.04243	0.51	0.9

**Table 2:** Alcoholic strength values obtained by electronic densimetry (A) and FTIR (B) and methanol concentrations obtained by GC-FID (C) and FTIR (D).

Wine Sample	Type	Alcoholic Strength (A) (% *v*/*v*)	Alcoholic Strength (B) (% *v*/*v*)	Methanol (C) (mg/L)	Methanol (D) (mg/L)
‘Alvarinho’ 2016	White	13.45 ± 0.01	13.37 ± 0.24	22.5 ± 0.7	250 ± 353.6
‘Alvarinho’ 2019	White	13.68 ± 0.02	12.66 ± 0.22	38.0 ± 9.9	150 ± 212.1
‘Arinto’ 2019	White	12.34 ± 0.00	11.59 ± 0.26	51.0 ± 4.2	55 ± 77.8
‘Cabernet Sauvignon’ 2016	Red	12.12 ± 0.01	14.2 ± 0.28	102.5± 6.4	400 ± 565.7
‘Cabernet Sauvignon’ 2018	Red	13.00 ± 0.01	13.49 ± 0.27	83.5 ± 9.2	80 ± 28.3
‘Cabernet Sauvignon’ 2019	Red	12.23 ± 0.01	11.27 ± 0.23	111.0 ± 2.8	200 ± 282.8
‘Encruzado’ 2019	White	12.96 ± 0.06	11.78 ± 0.25	23.5 ± 2.1	115 ± 120.2
‘Moscatel de Setúbal’ 2019	White	13.27 ± 0.01	12.96 ± 0.23	22.5 ± 2.1	45 ± 63.6
‘Macabeo’ 2019	White	10.99 ± 0.01	10.55 ± 0.21	75.0 ± 9.9	80 ± 113.1
‘Moscatel de Setúbal’ 2016	White	13.19 ± 0.02	10.06 ± 0.23	17.0 ± 1.4	60 ± 84.9
‘Moscatel Galego’ 2019	White	15.53 ± 0.05	15.89 ± 0.26	53.5 ± 7.8	300 ± 424.3
‘Syrah’ 2016	Red	15.25 ± 0.04	17.58 ± 0.25	174.5 ± 4.9	200 ± 282.8
‘Syrah’ 2018	Red	16.10 ± 0.02	14.69 ± 0.27	124.5 ± 3.5	135 ± 190.9
‘Syrah’ 2019	Red	16.93 ± 0.02	15.78 ± 0.25	135.0 ± 8.5	200 ± 282.8
‘Touriga Nacional’ 2016	Red	10.75 ± 0.01	12.57 ± 0.23	137.0 ± 8.5	150 ± 212.1
‘Touriga Nacional’ 2018	Red	16.28 ± 0.01	15.89 ± 0.26	172.5 ± 4.9	0 ± 0
‘Touriga Nacional’ 2019	Red	15.71 ± 0.01	12.06 ± 0.23	214.0 ± 32.5	150 ± 212.1
‘Trincadeira’ 2019	Red	13.58 ± 0.01	12.28 ± 0.25	211.5 ± 19.1	200 ± 282.8
‘Trincadeira’ 2016	Red	14.24 ± 0.00	15.11 ± 0.30	194.0 ± 29.7	400 ± 565.7
‘Viosinho’ 2019	White	13.45 ± 0.03	12.68 ± 0.46	21.5 ± 3.5	100 ± 141.4

## Data Availability

The original contributions presented in the study are included in the article, further inquiries can be directed to the corresponding author.
